# Leptospirosis Prevalence and Risk Factors Among Patients Presenting With Fever to 4 Healthcare Sites in Sub-Saharan Africa and South East Asia: An International Multisite Observational and Nested Case–Control Study

**DOI:** 10.1093/infdis/jiaf464

**Published:** 2025-10-16

**Authors:** John A Crump, Polycarp Mogeni, Sara A Ajanovic, Justina M Bramugy, Mabvuto Chimenya, Edward W Green, Sham Lal, David C W Mabey, Mayfong Mayxay, Paul N Newton, Ioana D Olaru, Heidi Hopkins, Mathieu Picardeau, Febrile Illness Evaluation in a Broad Range of Endemicities (FIEBRE) Consortium, Benjamin Amos, Elizabeth A Ashley, Oliver Baerenbold, Stéphanie Baghoumina, Núria Balanza, Tsitsi Bandason, Quique Bassat, Tapan Bhattacharyya, Stuart D Blacksell, Zumilda Boca, Christian Bottomley, John Bradley, Clare I R Chandler, Vilada Chansamouth, Joseph Chipanga, Anelsio Cossa, Ethel Dauya, Catherine Davis, Justin Dixon, Somyoth Douangphachanh, Audrey Dubot-Pérès, Michelle M Durkin, Nicholas A Feasey, Rashida A Ferrand, Colin Fink, Elizabeth J A Fitchett, Alessandro Gerada, Stephen R Graves, Becca L Handley, Coll D Hutchison, Risara Jaksuwan, Jessica Jervis, Jayne Jones, Kevin C Kain, Suzanne H Keddie, Khamxeng Khounpaseuth, Katharina Kranzer, Khamfong Kunlaya, Pankaj Lal, Xavier de Lamballerie, David G Lalloo, Manophab Luangraj, Yoel Lubell, Eleanor MacPherson, Sengchanh Manichan, Tegwen Marlais, Florian Maurer, Michael Miles, Campos Mucasse, Chelsea Nguyen, Vilayouth Phimolsarnnousith, Chrissy h Roberts, Amphone Sengduangphachanh, Siho Sengsavang, Molly Sibanda, Somvai Singha, John Stenos, Ampai Tanganuchitcharnchai, Hira Tanvir, James E Ussher, Marta Valente, Marie A Voice, Manivanh Vongsouvath, Msopole Wamaka, L Joseph Wheat, Shunmay Yeung

**Affiliations:** Centre for International Health, University of Otago, Dunedin, New Zealand; London School of Hygiene and Tropical Medicine, London, United Kingdom; Centro de Investigação em Saúde de Manhiça, Maputo, Mozambique; ISGlobal—Barcelona Institute for Global Health, Barcelona, Spain; University of Barcelona, Barcelona, Spain; Centro de Investigação em Saúde de Manhiça, Maputo, Mozambique; Malawi–Liverpool–Wellcome Trust Clinical Research Programme, Blantyre, Malawi; Malawi–Liverpool–Wellcome Trust Clinical Research Programme, Blantyre, Malawi; Department of Clinical Sciences, Liverpool School of Tropical Medicine, Liverpool, United Kingdom; London School of Hygiene and Tropical Medicine, London, United Kingdom; London School of Hygiene and Tropical Medicine, London, United Kingdom; Lao–Oxford–Mahosot Hospital–Wellcome Trust Research Unit (LOMWRU), Microbiology Laboratory, Mahosot Hospital, Vientiane, Lao PDR; Centre for Tropical Medicine and Global Health, Nuffield Department of Medicine, University of Oxford, Oxford, United Kingdom; Institute of Research and Education Development, University of Health Sciences, Ministry of Health, Vientiane, Lao PDR; Saw Swee Hock School of Public Health, National University of Singapore, Singapore; Lao–Oxford–Mahosot Hospital–Wellcome Trust Research Unit (LOMWRU), Microbiology Laboratory, Mahosot Hospital, Vientiane, Lao PDR; Centre for Tropical Medicine and Global Health, Nuffield Department of Medicine, University of Oxford, Oxford, United Kingdom; London School of Hygiene and Tropical Medicine, London, United Kingdom; Biomedical Research and Training Institute, Harare, Zimbabwe; Institute of Medical Microbiology, University Hospital Münster, Münster, Germany; London School of Hygiene and Tropical Medicine, London, United Kingdom; Unité Biologie des Spirochètes, French National Reference Center for Leptospirosis, WHO Collaborating Center for Reference and Research on Leptospirosis, Institut Pasteur, Paris, France

**Keywords:** fever, *Leptospira*, leptospirosis, prevalence, risk factors

## Abstract

**Background:**

We investigated the prevalence, diversity, and risk factors for acute leptospirosis in the Febrile Illness Evaluation in a Broad Range of Endemicities (FIEBRE) study.

**Methods:**

Febrile patients aged ≥2 months in Laos, Malawi, Mozambique, and Zimbabwe underwent a standardized clinical and exposure assessment. Acute and convalescent serum were tested by *Leptospira* microscopic agglutination test (MAT) and acute plasma by *lfb1* polymerase chain reaction. A ≥4-fold rise in antibody titer, or a single reciprocal titer ≥800, or *Leptospira* PCR positive defined confirmed leptospirosis. The identity of possible infecting strains was investigated by MAT and sequencing of PCR products.

**Results:**

Of 7851 febrile participants enrolled, 134 (1.7%) had confirmed leptospirosis: 88 (4.6%) in Laos, 17 (1.0%) Malawi, 7 (0.3%) Mozambique, and 22 (1.2%) Zimbabwe, and 23 (0.8%) had supportive evidence of leptospirosis. Participants with leptospirosis had greater odds of headache (adjusted odds ratio [aOR] 2.20, *P* < .001), rash (aOR 1.45, *P* < .001), conjunctivitis (aOR 3.33, *P* < .001), and jaundice (aOR 1.75, *P* < .001); and had greater odds of being older (aOR 1.02 per year, *P* < .001), working in rice fields (aOR 6.24, *P* < .001), drinking river water (aOR 5.11, *P* = .001). Predominant reactive *Leptospira* serogroups were Ballum and Icterohemorrhagiae at African sites, and Australis in Laos. Identified species were *Leptospira borgpetersenii*, *L. interrogans*, and *L. kirschneri*.

**Conclusions:**

Leptospirosis was a cause of febrile illness at all sites. Some clinical features helped to identify patients with leptospirosis. Interventions related to rice field work and river exposure may prevent disease. Diverse *Leptospira* serogroup reactivity was observed and may suggest potential hosts.

## Teaser

*Leptospira interrogans*, *Leptospira kirschneri*, or *Leptospira borgpetersenii* caused leptospirosis. Seroreactivity to *Leptospira* serogroups Ballum and Icterohemorrhagiae predominated at Africa sites and Australis in Laos. Lepospirosis was associated with rice field and river water exposure, with rats and cattle as potential hosts.

Leptospirosis is a zoonotic bacterial infection established as a major cause of fever in Asia [1] and increasingly recognized as an important cause of fever in Africa [2, 3]. Leptospirosis commonly presents as a febrile illness in humans that is difficult to distinguish clinically from other causes of fever and may be severe and fatal [4]. Reference standard laboratory diagnosis relies on microscopic agglutination testing (MAT) of paired sera, nucleic acid amplification tests, and bacterial isolation rarely available in low-resource endemic areas [5]. *Leptospira* includes diverse pathogenic and intermediate pathogenic strains capable of causing human illness across a wide range of *Leptospira* species, serogroups, and serovars. Nonhuman animals are the reservoirs of *Leptospira* spp. associated with human illness. Infected animals may remain well or experience a range of clinical manifestations from infertility, abortion, and poor milk yield, and become carriers, continuing to excrete *Leptospira* in their urine, in turn contaminating water and moist soil [5]. Humans are infected following direct contact with the urine of animals shedding *Leptospira* or indirectly by contacted with urine-contaminated environments [6]. The organism enters the human host via mucous membranes and broken skin. *Leptospira* have a complex and diverse ecology with variation in dominant host species by location and over time. Rodents are recognized as major reservoirs of *Leptospira* worldwide [5] and activities exposing persons to rodent urine, including residence in an urban slum, proximity to open sewers, exposure to floodwaters, and rice paddy work, are associated with increased risk for disease [5–7]. Risk factors for leptospirosis are less well understood in mainland Africa than elsewhere. While rodents are likely to be important hosts, cattle exposure was associated with increased odds for disease at some locations [8–11].

The lack of widespread access to reference diagnostic tests for leptospirosis, their complexity, and the limited investment in leptospirosis as a neglected tropical zoonosis mean that our understanding of its role as a cause of febrile illness and associated risk factors is incomplete [12, 13]. The Febrile Illness Evaluation in a Broad Range of Endemicities (FIEBRE) study [14] sought to describe treatable and preventable causes of febrile illness among inpatients and outpatients at multiple sites with little or no data on leptospirosis and other causes of fever using reference standard diagnostics.

To provide insights into variation in the role of acute leptospirosis as a cause of febrile illness, the diversity of infecting *Leptospira* species and serogroups, clinical features, clinical diagnosis, behaviors, and exposures associated with disease, we undertook a secondary analysis of FIEBRE data focused on participants with and without leptospirosis. We extended this work by examining the predominant reactive *Leptospira* serogroups associated with human illness in FIEBRE sites and their potential hosts.

## METHODS

The overall design of FIEBRE has been described in detail elsewhere [14]. A brief summary is provided below, including detailed methods for the present analysis.

### Study Sites

FIEBRE was conducted at 4 sites: Vientiane Provincial Hospital, Phonghong, Lao People's Democratic Republic (Laos); Chikwawa District Hospital, Malawi; Manhiça District Hospital and General Macamo Hospital, Maputo, Mozambique; and Sally Mugabe Central Hospital, Parirenyatwa Hospital, Chitungwiza General Hospital, and 3 primary care clinics in Harare, Zimbabwe.

### Enrollment, Follow-up, Data Collection, and Sample Collection for Febrile Participants

Febrile outpatients and inpatients aged ≥2 months were eligible for enrollment if they had a tympanic or axillary temperature of ≥37.5°C at presentation and had not been hospitalized or undergone surgery in the previous month. Outpatients were eligible if they were residents within a defined catchment area around the health facility at the time of enrollment. For outpatients aged ≥15 years, those without symptoms of either lower respiratory infection or diarrheal disease were eligible. For outpatients aged ≥2 months to <15 years, those without symptoms of diarrheal disease were eligible. After provision of informed consent, demographic information and a standardized clinical and exposure history were taken, and physical examination was performed on all participants and recorded on a case report form (Supplementary Appendix). The exposure history included questions relevant to risk for leptospirosis, such as exposure to surface water, rice fields, and animals, including livestock.

Whole blood and ethylenediaminetetraacetic acid (EDTA) blood were collected on day 0, and participants were asked to return 28 days after enrollment (acceptable range: 26–48 days) for collection of convalescent whole blood. Serum and plasma were separated, aliquoted, and stored at −70°C. Samples were shipped on dry ice to the London School of Hygiene and Tropical Medicine, London, United Kingdom, for distribution on dry ice to reference laboratories.

### Laboratory Methods

#### *Leptospira* Serology

At the Unité Biologie des Spirochètes, French National Reference Center for Leptospirosis, WHO Collaborating Centre for Reference and Research on Leptospirosis, Institut Pasteur, Paris, France, acute and convalescent serum was first screened by *Leptospira fainei* serovar Hurstbridge IgM enzyme-linked immunosorbent assay (ELISA) [15]. Serum of participants screening positive by ELISA on the acute or convalescent sample proceeded to testing with the *Leptospira* standard MAT. The MAT panels were based on the World Health Organization recommended list of globally representative reference *Leptospira* strains [16] adjusted to incorporate African and Asian regional isolates (Supplementary Appendix).

#### *Leptospira* Nucleic Acid Amplification Testing and Speciation

Acute plasma collected in EDTA from all participants was tested by polymerase chain reaction (PCR) to the *lfb1* pathogenic *Leptospira* target [17]. Among samples positive by *Leptospira lfb1* PCR, PCR products were sequenced for *Leptospira* speciation [18, 19].

### Study Definitions

For the purpose of this analysis and consistent with widely accepted case definitions [20], we defined confirmed acute leptospirosis as a participant with ≥4-fold rise in antibody titer between acute and convalescent sample or a single reciprocal titer ≥800, or *Leptospira* PCR positive for the *lfb1* gene target. We defined supportive evidence of leptospirosis as a participant with a single reciprocal titer ≥200 but <800. The predominant reactive serogroup was defined as the serogroup for the reacting serovar with the highest MAT titer.

### Potential Host Animal Species of *Leptospira*

To generate hypotheses about potential host animals of *Leptospira* by site, we interrogated the Dr Leopold Kirschner database of *Leptospira* species and serovar isolations and detections from animals worldwide at the serogroup level [21, 22]. We did this for leading predominant reactive *Leptospira* serogroups by study site for participants with serologically confirmed leptospirosis at the level of country and United Nations region.

### Sample Size

The sample size for this analysis was driven by that of the parent FIEBRE study and is described in detail elsewhere [14].

### Statistical Analyses

We used median and interquartile range (IQR) to summarize continuous variables, while categorical variables were summarized using percentages. We used range to summarize antibody titers by serogroup. To investigate clinical predictors and exposures associated with leptospirosis, we used separate logistic regression models to assess the associations between confirmed or supportive evidence of leptospirosis and the sociodemographic and clinical variables, adjusted for sex and age without data imputation. To account for the possibility of differences in predominant exposure pathways between Africa and Asia, we conducted subgroup analyses separately for data from African sites and from the Asian site. We used bivariable analyses, retaining significant covariates at *P* < .1 and backward exclusion of nonsignificant covariates with *P* > .05 to arrive at a final parsimonious model. The variance inflation factor was used to examine potential collinearity. Age and sex were considered a *priori* confounders and were included in multivariable models. Uncertainty was reported as 95% confidence intervals (CIs). To explore the possibility of variation in predominant transmission routes by continent, and particularly the role of ruminant livestock as sources for leptospirosis at African sites [8–10], we undertook subgroup analyses separately for data from Africa and Laos. All data analyses were performed using STATA version 18.0 (Stata Corp, College Station, Texas).

### Research Ethics

Ethics approval was obtained from the Lao National Ethics Committee for Health Research Committee and the Oxford Tropical Research Ethics Committee, United Kingdom, for Laos; the University of Malawi College of Medicine Research and Ethics Committee and the Liverpool School of Tropical Medicine Research Ethics Committee for Malawi; Comité Institucional de Medical Research Bioética para a Saúde do Centro de Investigação em Saúde de Manhiça and the Comité Nacional de Bioética em Saúde de Moçambique for Mozambique; and the Medical Research Council of Zimbabwe for Zimbabwe. The study was also approved by the research and ethics committee of the London School of Hygiene and Tropical Medicine. Written informed consent was obtained from all study participants or their parents or guardians.

### Role of the Funding Source

The funder had no role in study design; in the collection, analysis, and interpretation of data; in the writing of the report; nor in the decision to submit the paper for publication.

## RESULTS

**Figure 1.**
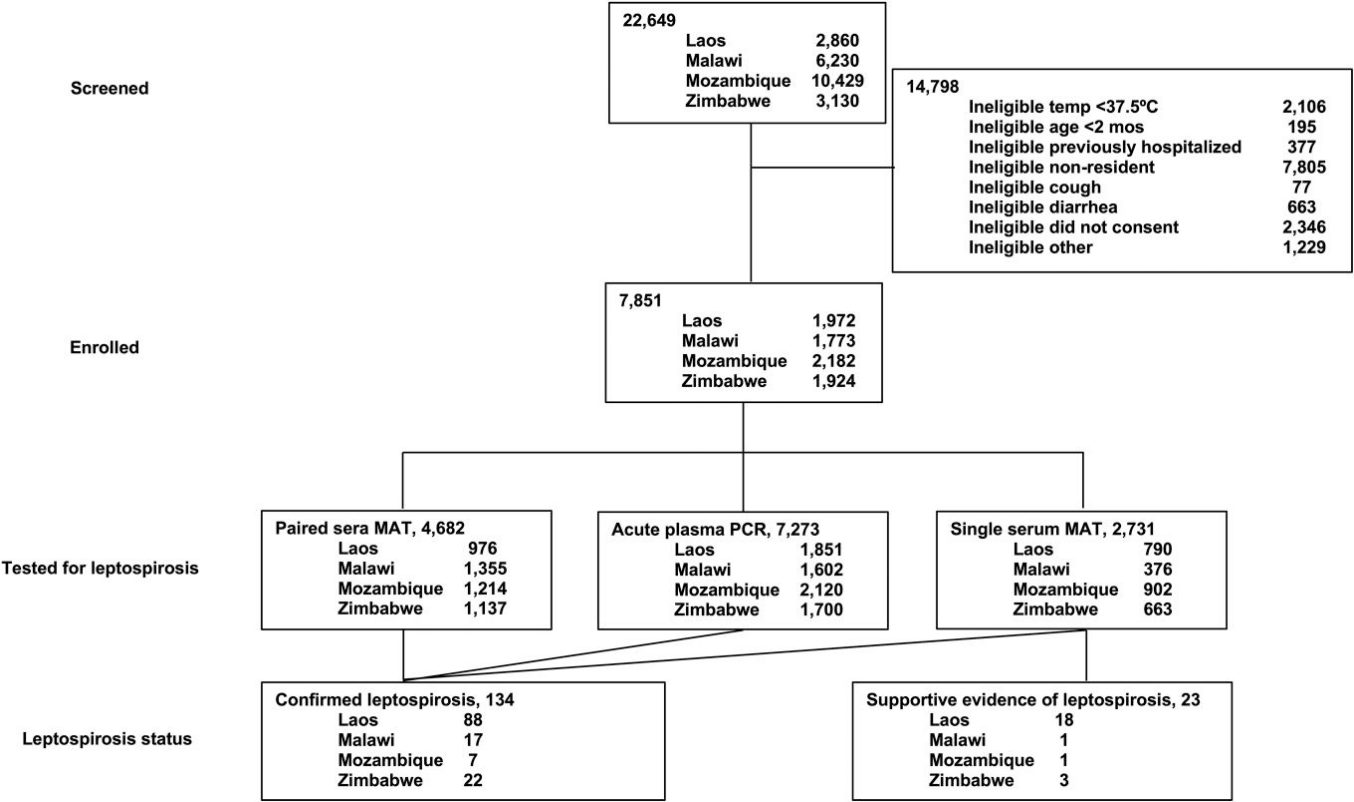
Study flow diagram for acute leptospirosis prevalence and risk factors in the Febrile Illness Evaluation in a Broad Range of Endemicities (FIEBRE) study, 2018–2021.

Of 7851 febrile participants enrolled across study sites from 22 June 2018 to 31 March 2021, 142 had missing serology and PCR results, and 7413 (94.4%) had at least day 0 or day 28 serum samples available for testing for leptospirosis, of which 4682 (59.6%) had paired day 0 and day 28 sera available, and 7273 (92.6%) had plasma available for PCR analysis (Figure 1).

Of 7851 febrile participants, 134 (1.7%) had confirmed leptospirosis: 89 (1.1%) by ≥4-fold rise in *Leptospira* MAT titer between the acute and convalescent sample, 13 (0.2%) by a single *Leptospira* MAT reciprocal titer ≥800, and 38 (0.5%) by *Leptospira lfb1* PCR. Among those confirmed by *Leptospira lfb1* PCR, the detected *Leptospira* species are shown in Table 1. Confirmed leptospirosis was identified in 88 (4.6%) participants from Laos, 177 (1.0%) from Malawi, 7 (0.3%) from Mozambique, and 22 (1.2%) from Zimbabwe (Table 2). Of 89 participants with a ≥4-fold rise in *Leptospira* MAT titer between the acute and convalescent sample and a *Leptospira lfb1* PCR result, 6 (6.7%) were also *Leptospira lfb1* PCR positive. Of 12 participants with a single *Leptospira* MAT reciprocal titer ≥800 and a *Leptospira lfb1* PCR result, 0 (0.0%) were *Leptospira lfb1* PCR positive. Of 22 participants positive by *Leptospira lfb1* PCR and paired sera tested by *Leptospira* MAT, 6 (27.3%) also had a ≥4-fold rise in *Leptospira* MAT titer between the acute and convalescent sample. Of 16 participants who were positive by *Leptospira lfb1* PCR and a serum sample tested by *Leptospira* MAT, 0 (0.0%) had a single *Leptospira* MAT reciprocal titer ≥800. In Laos 14 (15.9%) of 88 cases of confirmed leptospirosis were diagnosed by PCR, whereas in Africa sites 24 (52.2%) of 46 cases of confirmed leptospirosis were diagnosed by PCR.

**Table 1.** *Leptospira* Species Identified Among *lfb1* PCR–Positive Participants in the Febrile Illness Evaluation in a Broad Range of Endemicities (FIEBRE) Study, 2018–2021

Site	Species	N Positive Age <15 y	N Positive Age ≥15 y	N Positive All Ages
Laos	*L. interrogans*	3	10	13
	*L. kirschneri*	0	1	1
Malawi	*L. borgpetersenii*	0	1	1
	*L. interrogans*	2	3	5
Mozambique	*L. interrogans*	2	0	2
	*L. kirschneri*	0	4	4
Zimbabwe	*L. borgpetersenii*	0	2	2
	*L. interrogans*	6	4	10
Overall	*L. borgpetersenii*	0	3	3
	*L. interrogans*	13	17	30
	*L. kirschneri*	0	5	5

**Table 2.** Confirmed or Supportive Evidence of Leptospirosis by Study Site, Age Group, and Case Definition Criterion for Patients Presenting With Fever for Health Care in the Febrile Illness Evaluation in a Broad Range of Endemicities (FIEBRE) Study, 2018–2021

	Age Group, y	≥4-Fold Rise In *Leptospira* MAT Titer Between Paired Sera			Single *Leptospira* MAT Titer ≥800			*Lfb1* PCR Positive			Total Confirmed Leptospirosis			Single Reciprocal Titer ≥200 But <800			Total Confirmed or Supportive Evidence of Leptospirosis		
		N	n	(%)	N	n	(%)	N	n	(%)	N	n	(%)	N	n	(%)	N	n	(%)
Laos	<15	241	9	(3.73)	352	5	(1.42)	654	3	(0.46)	717	16	(2.23)	352	5	(1.42)	717	21	(2.93)
	≥15	735	59	(8.03)	438	4	(0.90)	1197	11	(0.92)	1206	72	(5.97)	438	13	(2.97)	1206	85	(7.05)
	All ages	976	68	(6.97)	790	9	(1.14)	1851	14	(0.76)	1923	88	(4.58)	790	18	(2.28)	1923	106	(5.51)
Malawi	<15	674	2	(0.30)	244	1	(0.41)	863	2	(0.23)	930	5	(0.54)	244	0	(0.00)	1020	5	(0.49)
	≥15	681	8	(1.17)	132	0	(0.00)	739	4	(0.54)	819	12	(0.54)	132	1	(0.76)	819	13	(1.59)
	All ages	1355	10	(0.74)	376	1	(0.27)	1602	6	(0.37)	1749	17	(1.47)	376	1	(0.27)	1749	18	(1.03)
Mozambique	<15	561	0	(0.00)	546	0	(0.00)	1122	2	(0.18)	1157	2	(0.17)	546	0	(0.00)	1157	2	(0.17)
	≥15	653	2	(0.31)	356	0	(0.00)	998	4	(0.40)	1016	5	(0.49)	356	1	(0.28)	1016	6	(0.59)
	All ages	1214	2	(0.16)	902	0	(0.00)	2120	6	(0.28)	2173	7	(0.32)	902	1	(0.11)	2173	8	(0.37)
Zimbabwe	<15	408	1	(0.25)	372	1	(0.27)	703	6	(0.85)	828	8	(0.97)	372	1	(0.27)	828	9	(1.09)
	≥15	729	8	(1.10)	291	2	(0.69)	997	6	(0.60)	1036	14	(1.35)	291	2	(0.69)	1036	16	(1.54)
	All ages	1137	9	(0.79)	663	3	(0.45)	1700	12	(0.71)	1864	22	(1.18)	663	3	(0.45)	1864	25	(1.34)
Total	<15	1844	12	(0.64)	1514	7	(0.46)	3342	13	(0.39)	3632	31	(0.85)	1514	6	(0.40)	3632	37	(1.02)
	≥15	2798	77	(2.75)	1217	6	(0.49)	3931	25	(0.64)	4077	103	(2.53)	1217	17	(1.40)	4077	120	(2.94)
	All ages	4682	89	(1.90)	2731	13	(0.48)	7273	38	(0.52)	7709	134	(1.75)	2731	23	(0.84)	7709	157	(2.04)

Abbreviations: Laos, Lao People's Democratic Republic; MAT, standard microscopic agglutination test; PCR, polymerase chain reaction.

The median (IQR) age of all participants with confirmed leptospirosis was 27 (17–41) years compared with 18 (4.8–33) years for those without confirmed leptospirosis (*P* < .001). Of pediatric participants, 31 (0.9%) had confirmed leptospirosis, and the median (IQR) age was 8 (5–11) years. Among adult participants, 103 (2.5%) had confirmed leptospirosis, and the median (IQR) age was 35 (23–46) years. Among all participants, 62 (46.3%) with confirmed leptospirosis were female compared with 4006 (52.9%) of those without confirmed leptospirosis (*P* = .128).

Of 7851 febrile participants, 23 (0.3%) had supportive evidence of leptospirosis. Supportive evidence of leptospirosis was identified in 18 (2.3%) participants from Laos, 1 (0.3%) from Malawi, 1 (0.1%) from Mozambique, and 3 (0.5%) from Zimbabwe (Table 2).

### Clinical Characteristics and Predictors of Leptospirosis

Clinical characteristics of participants with and without confirmed or supportive evidence of leptospirosis are shown in Table 3. On bivariable analysis of participants with confirmed or supportive evidence of leptospirosis compared with those without the median (IQR) admission temperature was 38.0°C (37.6°C, 38.5°C) versus 38.1°C (37.7°C, 38.9°C) (odds ratio [OR] 0.77 per °C, *P* = .026), median (IQR) oxygen saturation 96.0% (95.0%, 98.0%) versus 97.0% (95.0%, 98.0%) (OR 0.96 per %, *P* = .008), median (IQR) respiratory rate was 22.0 (20.0. 25.0) versus 22.0 (20.0, 28.0) per minute (OR 0.96 per breath per minute, *P* = .001), headache was reported by 114 (78.6%) versus 3440 (66.1%) (OR 1.9, *P* = .002), conjunctivitis was observed in 4 (2.5%) versus 57 (0.8%) (OR 3.40, *P* = .019), the median (IQR) Universal Vital Assessment score among adults was 0.0 (0.0–0.0) versus 0 (0.0–2.0) (OR per unit 0.65, *P* < .001), jaundice was seen in 10 (6.3%) versus 195 (2.6%) (OR 2.56, *P* = .005), and antimalarials were administered in 3 (1.9%) versus 980 (13.2%) (OR 0.13, *P* = .001). A multivariable analysis found headache (adjusted OR [aOR] 2.20, *P* < .001), rash or skin lesions (aOR 1.45, *P* = .001), conjunctivitis (aOR 3.33, *P* < .001), and jaundice (aOR 1.75, *P* < .001) remained associated with confirmed or supportive evidence of leptospirosis, whereas administration of antimalarials was associated with reduced odds of confirmed or supportive evidence of leptospirosis (aOR 0.10, *P* = .002). Of those with confirmed or supportive evidence of leptospirosis, 2 (1.3%) of 151 with vital outcome data died in hospital.

**Table 3.** Clinical Characteristics of Participants With and Without Confirmed Leptospirosis or Supportive Evidence of Leptospirosis by Bivariable and Logistic Regression, Febrile Illness Evaluation in a Broad Range of Endemicities (FIEBRE) Study, 2018–2021

	Confirmed or Supportive N = 157		Neither N = 7551		Unadjusted Model			Adjusted Model**		
	n	(%)	n	(%)	OR	95% CI	*P*-value	aOR	95% CI	*P*-value
Sex										
Female	75	(47.8)	3993	(52.9)	Ref.			Ref.	…	…
Male	82	(52.2)	3558	(47.1)	1.23	(.89,1.68)	.205	1.33	(.75, 2.38)	.332
Age years, median (IQR)	27.0	(17.0–43.0)	18.0	(4.7–33.0)	1.02	(1.01, 1.03)	<.001	1.01	(1.00, 1.02)	.107
Health facility admission status										
Outpatient	80	(51.0)	4320	(57.2)	Ref.	…		Ref.	…	…
Inpatient	77	(49.0)	3232	(42.8)	1.29	(.94, 1.76)	.118	1.43	(.75, 2.74)	.278
Admission temperature °C, median (IQR)	38.0	(37.6–38.5)	38.1	(37.7–38.9)	0.77	(.61, .97)	.026	…	…	…
Systolic blood pressure mmHg, median (IQR)	110.0	(100.0–120.0)	110.0	(97.0–120.0)	1.00	(1.00, 1.00)	.990	…	…	…
Diastolic blood pressure mmHg, median (IQR)	70.0	(60.0–80.0)	70.0	(60.0–79.0)	0.99	(.98, 1.01)	.384	…	…	…
O_2_ saturation %, median (IQR)	96.0	(95.0–98.0)	97.0	(95.0–98.0)	0.97	(.94, .99)	.008	…	…	…
Respiratory rate per minute, median (IQR)	22.0	(20.0–25.0)	22.0	(20.0–28.0)	0.96	(.93, .98)	.001	…	…	…
Diarrhea										
No	33	(89.2)	3290	(91.8)	Ref.	…		…	…	…
Yes	4	(10.8)	292	(8.2)	1.37	(.48, 3.88)	.559	…	…	…
History of fever days, median (IQR)	3.0	(2.0–4.0)	2.0	(2.0–4.0)	1.00	(.97, 1.04)	.914	…	…	…
Cough										
No	108	(68.8)	4847	(64.7)	Ref.	…		…	…	…
Yes	49	(31.2)	2639	(35.3)	0.83	(.59, 1.17)	.294	…	…	…
Vomit										
No	128	(81.5)	6372	(85.1)	…	…		…	…	…
Yes	29	(18.5)	1115	(14.9)	1.29	(.86, 1.95)	.215	…	…	…
Headache										
No	31	(21.4)	1765	(33.9)	Ref.	…		Ref.	…	…
Yes	114	(78.6)	3440	(66.1)	1.89	(1.26, 2.82)	.002	2.20	(1.79, 2.70)	<.001
Musculoskeletal pain										
No	59	(49.2)	1995	(51.4)	Ref.	…		…	…	…
Yes	61	(50.8)	1890	(48.6)	1.09	(.76, 1.57)	.637	…	…	…
Abdominal pain										
No	118	(75.6)	5588	(76.6)	Ref.	…		…	…	…
Yes	38	(24.4)	1708	(23.4)	1.05	(.73, 1.52)	.782	…	…	…
Rash or skin lesions										
No	144	(91.7)	7072	(94.0)	Ref.	…		Ref.	…	…
Yes	13	(8.2)	449	(6.0)	1.42	(.80, 2.53)	.231	1.45	(1.16, 1.82)	.001
Skin blanching										
No	13	(100.0)	430	(96.6)	Ref.	…		…	…	…
Yes	0	(0.0)	15	(3.4)	1.00	…		…	…	…
Conjunctivitis										
No	153	(97.5)	7462	(99.2)	Ref.	…		Ref.	…	…
Yes	4	(2.5)	57	(0.8)	3.42	(1.23, 9.55)	.019	3.33	(2.55, 4.36)	<.001
Jaundice										
No	147	(93.6)	7329	(97.4)	Ref.	…		Ref.	…	…
Yes	10	(6.4)	195	(2.6)	2.56	(1.33, 4.93)	.005	1.75	(1.39, 2.19)	<.001
Pediatric Early Warning Score, median (IQR)	14.0	(10.0–20.0)	11.0	(7.0–18.0)	1.01	(.98, 1.05)	.512	…	…	…
Universal Vital Assessment Score, median (IQR)	0.0	(0.0–0.0)	0.0	(0.0–2.0)	0.65	(.52, .81)	<.001	…	…	…
Logistic Organ Dysfunction Score, median (IQR)	0.0	(0.0–0.0)	0.0	(0.0–0.0)	0.53	(.13, 2.08)	.361	…	…	…
Antimalarials administered										
No	153	(98.1)	6463	(86.8)	Ref.	…		Ref.	…	…
Yes	3	(1.9)	980	(13.2)	0.13	(.04, .41)	<.001	0.10	(.02, .44)	.002
Any antimicrobial administered										
No	10	(76.9)	751	(88.2)	Ref.	…		…	…	…
Yes	3	(23.1)	100	(11.8)	2.25	(.61, 8.32)	.223	…	…	…
Died by day 28^a^										
No	136	(88.9)	6293	(85.8)	Ref.	…		…	…	…
Yes	5	(3.3)	266	(3.6)	0.87	(.35, 2.14)	.761	…	…	…
Lost to follow-up^b^	12	(7.8)	779	(10.6)	0.71	(.39, 1.29)	.265	…	…	…
Died in hospital^c^										
No	149	(94.9)	7279	(96.4)	Ref.	…		…	…	…
Yes	2	(1.3)	98	(1.3)	1.00	(.24, 4.08)	.997	…	…	…
Referred^d^	6	(3.8)	175	(2.3)	1.67	(.73, 3.84)	.223	…	…	…

Notes. FIEBRE enrollment criteria excluded some potential participants with diarrhea and lower respiratory infection syndromes, as described in the Methods section. Multivariable models were adjusted for age and sex, regardless of significance in the univariable regression models. Pediatric Early Warning Score, Universal Vital Assessment score, and Logistic Organ Dysfunction Score are not available for children and adults only, respectively.

Abbreviations: OR, odds ratio; aOR, adjusted odds ratio; 95% CI, 95% confidence interval; Ref., referent; IQR, interquartile range.

**Multivariable models were adjusted for age and sex, regardless of significance in the univariable regression models.

^a^Any death within 28 days of enrollment.

^b^Could not be traced at day 28 after enrollment.

^c^Any death among an inpatient participant occurring during the enrollment admission.

^d^In-hospital death status among inpatient participants could not be ascertained due to transfer to a nonstudy healthcare facility.

### Exposures Associated With Leptospirosis

Exposures reported by participants with and without confirmed or supportive evidence of leptospirosis are shown in Table 4. On bivariable analysis of participants with confirmed or supportive evidence of leptospirosis compared with those without the median (IQR) age was 26.5 (17.0, 43.0) versus 18.0 (4.7, 33.0) years (OR 1.02 per year, *P* < .001), living or working in close contact with cattle in the past month was reported by 19 (12.2%) versus 439 (5.9%) (OR 2.23, *P* = .001), living or working in close contact with cattle, goats, or pigs in the past month by 24 (15.4%) versus 769 (10.3%) (OR 1.59, *P* = .040), wading, swimming, or bathing in pond water, lake water, or steam water in the past month by 44 (28.0%) versus 667 (8.9%) (OR 3.98, *P* < .001), working in rice fields in the past month by 61 (38.9%) versus 580 (7.8%) (OR 7.56, *P* < .001), a river as the source of drinking water in the dry season by 3 (1.9%) versus 21 (0.3%) (OR 6.95, *P* = .002), and a river or pond the source of drinking water in the dry season by 3 (1.9%) versus 23 (0.3%) (OR 6.34, *P* = .003), respectively. On multivariable analysis, working in rice fields in the past month (aOR 6.24, *P* < .001) and having a river as the source of drinking water in the dry season (aOR 5.11, *P* = .001) remained associated with confirmed or supportive evidence of leptospirosis.

**Table 4.** Exposures Among Participants With and Without Confirmed Leptospirosis or Supportive Evidence of Leptospirosis by Bivariable and Logistic Regression, Febrile Illness Evaluation in a Broad Range of Endemicities (FIEBRE) Study, 2018–2021

	Confirmed Or Supportive N = 157		Neither N = 7551		Unadjusted Model			Adjusted Model**		
	n	(%)	n	(%)	OR	95% CI	*P*-value	aOR	95% CI	*P*-value
Sex										
Female	75	(47.8)	3993	(52.9)	Ref.	…		Ref.	…	…
Male	82	(52.2)	3558	(47.1)	1.23	(.89, 1.68)	.205	1.09	(.61, 1.94)	.772
Age years, median (IQR)	26.5	(17.0–43.0)	18.0	(4.7–33.0)	1.02	(1.01, 1.03)	<.001	1.02	(1.01, 1.02)	<.001
Lived or worked in close contact with cattle in the past month										
No	137	(87.8)	7048	(94.1)	…	…		…	…	…
Yes	19	(12.2)	439	(5.9)	2.23	(1.36, 3.63)	.001	…	…	…
Lived or worked in close contact with goats in the past month										
No	153	(97.5)	7105	(94.8)	Ref.	…		…	…	…
Yes	4	(2.5)	391	(5.2)	0.48	(.18, 1.29)	.144	…	…	…
Lived or worked in close contact with pigs in the past month										
No	149	(94.9)	7291	(97.2)	…	…		…	…	…
Yes	8	(5.1)	207	(2.8)	1.89	(.92, 3.90)	.085	…	…	…
Lived or worked in close contact with cattle, goats, or pigs in the past month										
No	132	(84.6)	6710	(89.7)	Ref.	…		…	…	…
Yes	24	(15.4)	769	(10.3)	1.59	(1.02, 2.47)	.040	…	…	…
Waded, swum or bathed in pond water, lake water, or stream water in the past month										
No	113	(72.0)	6822	(91.1)	Ref.	…		…	…	…
Yes	44	(28.0)	667	(8.9)	3.98	(2.79, 5.69)	<.001	…	…	…
Worked in rice fields in the past month										
No	96	(61.1)	6903	(92.2)	Ref.	…		Ref.	…	…
Yes	61	(38.9)	580	(7.8)	7.56	(5.43, 10.54)	<.001	6.24	(3.25, 11.98)	<.001
Source of drinking water in the dry season: river										
No	154	(98.1)	7489	(99.7)	Ref.	…		Ref.	…	…
Yes	3	(1.9)	21	(0.3)	6.95	(2.05, 23.54)	.002	5.11	(2.00, 13.09)	.001
Source of drinking water in the dry season: pond										
No	157	(100.0)	7508	(100.0)	Ref.	…		…	…	…
Yes	0	(0.0)	2	(<0.1)	1.00	…		…	…	…
Source of drinking water in the dry season: river or pond										
No	154	(98.1)	7487	(99.7)	Ref.	…		…	…	…
Yes	3	(1.9)	23	(0.3)	6.34	(1.88, 21.34)	.003	…	…	…

Notes. Multivariable models were adjusted for age and sex regardless of significance in the univariable regression models.

Abbreviations: OR, odds ratio; aOR, adjusted odds ratio; 95% CI, 95% confidence interval; Ref., referent; IQR, interquartile range.

**Multivariable models were adjusted for age and sex, regardless of significance in the univariable regression models.

Of participants at African sites, 4 (8.5%) of 47 with confirmed or supportive evidence of leptospirosis and 80 (1.4%) of 5673 without worked in rice fields. On multivariable analysis of exposures at African sites, working in rice fields in the past month (aOR 9.10, *P* < .001) was associated with confirmed or supportive evidence of leptospirosis, and living and working in close contact with cattle in the past month (aOR 1.56, *P* = .309) was not. In contrast, among participants at the Asian site, 47 (53.4%) of 88 with confirmed or supportive evidence of leptospirosis and 500 (27.6%) of 1809 without worked in rice fields. On multivariable analysis of exposures at the Laos site, working in rice fields in the past month (aOR 2.94, *P* = .001) and having a river as the source of drinking water in the dry season (aOR 9.25, *P* = .009) were associated with confirmed or supportive evidence of leptospirosis.

### Predominant Reactive *Leptospira* Serogroups, Species, and Potential Host Range

Table 5 shows the predominant reactive *Leptospira* serogroup among participants with serologically confirmed leptospirosis based on ≥4-fold rise in titer between acute and convalescent serum by study site. In Laos, participants with serologically confirmed leptospirosis were predominantly reactive to *Leptospira* serogroups Australis, Sejroe, Bataviae, Icterohemorrhagiae, Grippotyphosa, Autumnalis, Celledoni, Pomona, and Hebdomadis in descending order of prevalence. Strains representing these serogroups have been isolated from a wide range of host species in Asia, including but not limited to rodents, ruminant livestock, dogs, and pigs [21, 22]. In Malawi, participants with serologically confirmed leptospirosis were predominantly reactive to *Leptospira* serogroups Icterohemorrhagiae, Sejroe, Australis, Mini, and Pyrogenes; in Mozambique to *Leptospira* serogroups Australis, Autumnalis, and Icterohemorrhagiae; and in Zimbabwe to *Leptospira* serogroups Ballum, Icterohemorrhagiae, Pomona, and Pyrogenes. Strains representing these serogroups have been predominantly isolated from rats and cattle in Africa (Table 5) [21, 22]. Among the identified *Leptospira* species *Leptospira borgpetersenii*, *Leptospira interrogans*, and *Leptospira kirschneri*, variation was observed between study sites, also consistent with variation in host species (Table 1).

**Table 5.** Predominant Reactive Serogroup and Potential Regional *Leptospira* Reservoirs Among Participants With Serologically Confirmed Leptospirosis Based on ≥4-Fold Rise in Titer Between Acute and Convalescent Serum, Febrile Illness Evaluation in a Broad Range of Endemicities (FIEBRE) Study, 2018–2021

Site	Serogroup	N Positive	Acute Titer Range	Convalescent Titer Range	Median Titer	Potential Regional Reservoirs^a^
Laos	Australis	19	0–200	200–12 800	400	Cattle, rat, dog, pig, horse, nutria, toad
	Sejroe	17	0–200	200–3200	400	Cattle, dog, rat, vole
	Bataviae	14	0–200	200–1600	400	Rat, frog, cat
	Icterohemorrhagiae	12	0–200	200–6400	800	Rat, cattle, dog, pig, vole, civet, raccoon, mouse
	Grippotyphosa	9	0–0	200–1600	200	Rat, frog, cattle, dog, flying squirrel, goat, mouse, hedgehog, sheep, tick from cattle
	Autumnalis	3	0–0	800–1600	1600	Rat, cattle, dog, toad, hedgehog, pig
	Celledoni	3	0–0	400–12 800	800	Rat
	Pomona	2	0–0	400–800	600	Civet, cattle, mouse, mongoose, cat, dog, pig, rat, squirrel, fox
	Hebdomadis	1	0–0	200	200	Cattle, mouse, rat, raccoon, vole
Malawi	Icterohemorrhagiae	5	0–0	200–6400	200	Rat, cattle, mongoose, fox
	Sejroe	2	0–0	400–6400	3400	Rat, cattle
	Australis	1	0–0	6400	6400	Rat, cattle
	Mini	1	0–0	200	200	None
	Pyrogenes	1	0–0	200	200	Rat, cattle
Mozambique	Australis	2	0–0	200–200	200	Rat, cattle
	Autumnalis	1	0–0	200	200	Rat
	Icterohemorrhagiae	1	0–0	200	200	Rat, cattle, mongoose, fox
Zimbabwe	Ballum	6	0–0	200–6400	300	Rat, mouse
	Icterohemorrhagiae	3	0–0	200–800	400	Rat, cattle, mongoose, fox
	Pomona	1	0–0	200	200	Cattle
	Pyrogenes	1	0–0	200	200	Rat, cattle

^a^References by host are available in Dr Leopold Kirschner’s database [21, 22].

## DISCUSSION

Using a standardized study design and laboratory strategy, we showed that leptospirosis was a common cause of febrile illness at a site in Laos, and was present but less common at sites in Malawi, Mozambique, and Zimbabwe. In terms of clinical features, headache, rash or skin lesions, conjunctivitis, and jaundice were associated with increased odds of confirmed or supportive evidence of leptospirosis. Exposures, including working in rice fields in the past month and having a river as the source of drinking water in the dry season, were associated with increased odds of confirmed or supportive evidence of leptospirosis. *L. interrogans*, *L. kirschneri*, and *L. borgpetersenii* were confirmed in participants with positive *lfb1* PCR. A wider diversity of *Leptospira* predominant reactive serogroups and potential host species was observed in Laos compared with in African sites. Rats and cattle feature as common potential hosts across all study sites.

Leptospirosis often presents as a nonspecific febrile illness that is difficult to distinguish from other causes of fever by clinical history and physical examination alone [5]. We found that headache, rash or skin lesions, conjunctivitis, and jaundice, all recognized clinical features of leptospirosis, were associated with increased odds of the disease. However, while these features support a diagnosis of leptospirosis, none is sufficiently sensitive nor specific to be reliable for leptospirosis diagnosis alone. This highlights the need for wider access to reference diagnostics for leptospirosis, and better still, for developing point-of-care tests of sufficient accuracy to be useful for patient care [23, 24].

Working in rice fields in the past month and measures of surface water exposure were associated with increased odds of leptospirosis across FIEBRE study sites. Notably, an analysis of risk factors for leptospirosis restricted to African sites confirmed the role of rice field exposure at those sites but found no significant association with living and working in close contact with cattle despite other African studies identifying an important role for cattle exposure in leptospirosis risk [8, 10]. While rice field exposure was considerably less common among participants at African sites compared with the Asian site, this finding highlights the potential impact on leptospirosis risk of increases in irrigated rice farming in Africa [25]. The lack of association of leptospirosis with cattle exposure in African sites highlights the diverse ecology of the disease by location, even within the same region.

The diversity of *Leptospira* predominant reactive serogroups was higher in Laos than in African sites, possibly in part because the higher number of participants with serologically confirmed disease with serologically confirmed disease in Laos. Additionally, the Laos MAT panel was supplemented with local *Leptospira* strains, whereas we had limited ability to enrich the African MAT panel with local strains since relatively very few *Leptospira* strains have been isolated from African countries [21, 22]. This was in turn associated with a wider range of potential host species in Laos than in the African sites. While using predominant patterns of *Leptospira* serogroup reactivity provides only a crude insight into potential animal hosts of infecting strains [26], the findings generate hypotheses for future studies in these locations to investigate specific animal contacts as risk factors for human infection.

Our study had a number of limitations. MAT was only done after screening of acute and convalescent serum by *L. fainei* serovar Hurstbridge IgM ELISA, the sensitivity of which is uncertain for the diagnosis of leptospirosis particularly in Africa. Since the case definition for confirmed leptospirosis relied in part on the availability of a convalescent serum sample for MAT, it is likely that the case fatality ratio of leptospirosis was underestimated among those who died before a convalescent sample was collected. PCR to *lfb1* detected a larger proportion of confirmed leptospirosis infections relative to MAT among participants enrolled at African sites relative to the Asian site. This may reflect unmeasured differences in pre-enrollment antimicrobial use in Laos compared with the African sites, or perhaps the African MAT panel did not sufficiently represent the diversity of *Leptospira* strains circulating at the African sites compared with the Asian site. More work is needed to increase available isolates of pathogenic and intermediate pathogenic *Leptospira* from African countries to improve the performance of serologic diagnosis and gain a more complete picture of predominant reactive serogroup diversity. While our risk factor questionnaire included questions relevant to leptospirosis, it was not comprehensive and was insufficient to gain a nuanced picture of leptospirosis risk factors by site. The use as controls of participants presenting to healthcare facilities rather than those from the community may have introduced bias. Since leptospirosis was less common in Malawi, Mozambique, and Zimbabwe than Laos, our power to detect risk factors at African sites was reduced.

We confirm that leptospirosis is an important cause of fever in Laos and was present but less common at all 3 African sites studied. Since a specific diagnosis of leptospirosis cannot be made by clinical means alone, ongoing efforts to develop accurate diagnostic tests suitable for use in low-resource settings are needed. Rice field exposure is a major risk factor for infection, even in Africa, where rice field exposure was not as common, highlighting the need for focused prevention in rice field workers, especially as irrigated rice farming expands in Africa. We highlight the need to redouble efforts to isolate *Leptospira* strains in Africa to improve serologic diagnosis and identify hypotheses for more detailed risk factor studies.

## Supplementary Material

jiaf464_Supplementary_Data

## Supplementary Data

Supplementary materials are available at *The Journal of Infectious Diseases* online (http://jid.oxfordjournals.org/). Supplementary materials consist of data provided by the author that are published to benefit the reader. The posted materials are not copyedited. The contents of all supplementary data are the sole responsibility of the authors. Questions or messages regarding errors should be addressed to the author.
