# Supplementary material for: Leptospirosis Prevalence and Risk Factors Among Patients Presenting With Fever to 4 Healthcare Sites in Sub-Saharan Africa and South East Asia: An International Multisite Observational and Nested Case–Control Study
Source: J Infect Dis. 2025 Oct 16;233(1):e259–70. doi: 10.1093/infdis/jiaf464 (PMC12811888; doi:10.1093/infdis/jiaf464)
Supplement: jiaf464_Supplementary_Data [file jiaf464_supplementary_data.zip › Lepto_FIEBRE_Descriptive_SupplAppendix2_R1_15Aug2025.docx]

**Supplementary Table. Clinical characteristics of participants with and without confirmed leptospirosis by bivariable and logistic regression, Febrile Illness Evaluation in a Broad Range of Endemicities (FIEBRE) study, 2018-2021**

|  | **Confirmed**  **n = 134** | | **Not confirmed**  **n = 7,575** | | **Unadjusted model** | | | **Adjusted model**** | | |
| --- | --- | --- | --- | --- | --- | --- | --- | --- | --- | --- |
|  | **n** | **(%)** | **n** | **(%)** | **OR** | **95% CI** | **p-value** | **aOR** | **95% CI** | **p-value** |
| Sex |  |  |  |  |  |  |  |  |  |  |
| Female | 62 | (46.3) | 4,006 | (52.9) | Ref |  |  | Ref |  |  |
| Male | 72 | (53.7) | 3,568 | (47.1) | 1.30 | (0.93, 1.84) | 0.129 | 1.43 | (0.99, 2.05) | 0.054 |
| Age years, median (IQR) | 27.0 | (17.0-41.0) | 18.0 | (4.8-33.0) | 1.02 | (1.01, 1.03) | <0.001 | 1.01 | (1.00, 1.02) | 0.145 |
| Health facility admission status |  |  |  |  |  |  |  |  |  |  |
| Outpatient | 64 | (47.8) | 3,245 | (42.8) | Ref |  |  | Ref |  |  |
| Inpatient | 70 | (52.2) | 4,330 | (57.2) | 1.22 | (0.87, 1.72) | 0.254 |  |  |  |
| Admission temperature ºC, median (IQR) | 38.0 | (37.6-38.5) | 38.1 | (37.7-38.9) | 0.72 | (0.55, 0.93) | 0.011 |  |  |  |
| Systolic blood pressure mmHg, median (IQR) | 111.5 | (100.0-121.0) | 110.0 | (97.0-120.0) | 1.00 | (1.00, 1.00) | 0.875 |  |  |  |
| Diastolic blood pressure mmHg, median (IQR) | 70.0 | (60.0-80.0) | 70.0 | (60.0-79.0) | 1.00 | (0.90, 1.01) | 0.521 |  |  |  |
| O_2_ saturation %, median (IQR) | 96.0 | (95.0-98.0) | 97.0 | (95.0-98.0) | 0.96 | (0.94, 0.99) | 0.008 |  |  |  |
| Respiratory rate per minute, median (IQR) | 22.0 | (20.0-25.0) | 22.0 | (20.0-28.0) | 0.96 | (0.93, 0.99) | 0.003 |  |  |  |
| Diarrhoea |  |  |  |  |  |  |  |  |  |  |
| No | 27 | (87.1) | 3,296 | (91.9) | Ref |  |  |  |  |  |
| Yes | 4 | (12.9) | 292 | (8.1) | 1.67 | (0.58, 4.81) | 0.340 |  |  |  |
| History of fever days, median (IQR) | 3.0 | (2.0-4.0) | 2.0 | (2.0-4.0) | 1.00 | (0.97, 1.04) | 0.797 |  |  |  |
| Cough |  |  |  |  |  |  |  |  |  |  |
| No | 93 | (69.4) | 4,862 | (64.7) | Ref |  |  |  |  |  |
| Yes | 41 | (30.6) | 2,647 | (35.3) | 0.81 | (0.56, 1.17) | 0.264 |  |  |  |
| Vomit |  |  |  |  |  |  |  |  |  |  |
| No | 110 | (82.1) | 6,390 | (85.1) |  |  |  |  |  |  |
| Yes | 24 | (17.9) | 1,120 | (14.9) | 1.24 | (0.80, 1.94) | 0.336 |  |  |  |
| Headache |  |  |  |  |  |  |  |  |  |  |
| No | 25 | (20.2) | 1,771 | (33.9) | Ref |  |  | Ref |  |  |
| Yes | 99 | (79.8) | 3,445 | (66.1) | 2.03 | (1.30, 3.16) | 0.002 | 2.24 | (1.43, 3.51) | <0.001 |
| Musculoskeletal pain |  |  |  |  |  |  |  |  |  |  |
| No | 50 | (48.5) | 2,004 | (51.4) | Ref |  |  |  |  |  |
| Yes | 53 | (51.5) | 1,898 | (48.6) | 1.12 | (0.76, 1.66) | 0.573 |  |  |  |
| Abdominal pain |  |  |  |  |  |  |  |  |  |  |
| No | 101 | (75.4) | 5,605 | (76.6) | Ref |  |  |  |  |  |
| Yes | 33 | (24.6) | 1,713 | (23.4) | 1.07 | (0.72, 1.59) | 0.741 |  |  |  |
| Rash or skin lesions |  |  |  |  |  |  |  |  |  |  |
| No | 122 | (91.0) | 7,094 | (94.0) | Ref |  |  | Ref |  |  |
| Yes | 12 | (9.0) | 450 | (6.0) | 1.55 | (0.85, 2.83) | 0.152 | 1.70 | (0.91, 3.15) | 0.094 |
| Skin blanching |  |  |  |  |  |  |  |  |  |  |
| No | 12 | (100.0) | 431 | (96.6) | Ref |  |  |  |  |  |
| Yes | 0 | (0.0) | 15 | (3.4) | 1.00 |  |  |  |  |  |
| Conjunctivitis |  |  |  |  |  |  |  |  |  |  |
| No | 130 | (97.0) | 7,485 | (99.2) | Ref |  |  | Ref |  |  |
| Yes | 4 | (3.0) | 57 | (0.8) | 4.04 | (1.44,11.30) | 0.008 | 4.09 | (1.41,11.84) | 0.009 |
| Jaundice |  |  |  |  |  |  |  |  |  |  |
| No | 127 | (94.8) | 7,349 | (97.4) | Ref |  |  | Ref |  |  |
| Yes | 7 | (5.2) | 198 | (2.6) | 2.05 | (0.94, 4.44) | 0.070 | 1.43 | (0.62, 3.33) | 0.401 |
| Pediatric Early Warning Score, median (IQR) | 14.0 | (10.0-20.0) | 11.0 | (7.0-18.0) | 1.02 | (0.98, 1.06) | 0.397 |  |  |  |
| Universal Vital Assessment score, median (IQR) | 0.0 | (0.0-0.0) | 0.0 | (0.0-2.0) | 0.64 | (0.50, 0.81) | <0.001 |  |  |  |
| Logistic Organ Dysfunction Score, median (IQR) | 0.0 | (0.0-0.0) | 0.0 | (0.0-0.0) | 0.31 | (0.04, 2.17) | 0.238 |  |  |  |
| Antimalarials administered |  |  |  |  |  |  |  |  |  |  |
| No | 130 | (97.7) | 6,486 | (86.9) | Ref |  |  | Ref |  |  |
| Yes | 3 | (2.3) | 980 | (13.1) | 0.15 | (0.05, 0.48) | 0.001 | 0.13 | (0.03, 0.51) | 0.004 |
| Any antimicrobial administered |  |  |  |  |  |  |  |  |  |  |
| No | 10 | (76.9) | 751 | (88.2) | Ref |  |  |  |  |  |
| Yes | 3 | (23.1) | 100 | (11.8) | 2.25 | (0.61, 8.32) | 0.223 |  |  |  |
| Died by day 28* |  |  |  |  |  |  |  |  |  |  |
| No | 120 | (92.3) | 6,309 | (85.7) | Ref |  |  |  |  |  |
| Yes | 2 | (1.5) | 269 | (3.7) | 0.39 | (0.10, 1.59) | 0.189 |  |  |  |
| Lost to follow-up† | 8 | (6.2) | 783 | (10.6) | 0.54 | (0.26, 1.10) | 0.090 |  |  |  |
| Died in hospital‡ |  |  |  |  |  |  |  |  |  |  |
| No | 129 | (96.3) | 7,299 | (96.4) | Ref |  |  |  |  |  |
| Yes | 1 | (0.7) | 99 | (1.3) | 0.57 | (0.08, 4.13) | 0.579 |  |  |  |
| Referred§ | 4 | (3.0) | 177 | (2.3) | 1.28 | (0.47, 3.50) | 0.632 |  |  |  |

OR: odds ratio; aOR: adjusted odds ratio; 95% CI: 95% confidence interval; Ref: referent; IQR: interquartile range

*Any death within 28 days of enrolment; †Could not be traced at day 28 after enrolment; ‡Any death among an inpatient participant occurring during the enrolment admission; §In-hospital death status among inpatient participant could not ascertained due to transfer to a non-study healthcare facility.

FIEBRE enrolment criteria excluded some potential participants with diarrhea and lower respiratory infection syndromes, as described in the Methods.

Multivariable models were adjusted for age and sex, regardless of significance in the univariable regression models.

Pediatric Early Warning Score and Universal Vital Assessment score, and Logistic Organ Dysfunction Score, are not available for children and adults only, respectively.
